# Effects of Dietary Acute Tryptophan Depletion (ATD) on NPY Serum Levels in Healthy Adult Humans Whilst Controlling for Methionine Supply—A Pilot Study

**DOI:** 10.3390/nu10050594

**Published:** 2018-05-11

**Authors:** Janice W. Y. Wong, Hugo A. E. Morandini, Vita L. S. Dingerkus, Tilman J. Gaber, Kevin C. Runions, Pradeep Rao, Simone Mahfouda, Katrin Helmbold, Sarah Bubenzer-Busch, Rebecca Koenemann, Richard M. Stewart, Florian D. Zepf

**Affiliations:** 1 Centre & Discipline of Child and Adolescent Psychiatry, Psychosomatics and Psychotherapy, School of Medicine, Division of Psychiatry and Clinical Neurosciences and Division of Paediatrics and Child Health, The University of Western Australia, 6009 Perth, Australia; janice.wong@telethonkids.org.au (J.W.Y.W.); hugo.morandini@health.wa.gov.au (H.A.E.M.); kevin.runions@health.wa.gov.au (K.C.R.); pradeep.rao@health.wa.gov.au (P.R.); simone.mahfouda@telethonkids.org.au (S.M.); richard.stewart@health.wa.gov.au (R.M.S.); 2 Specialised Child and Adolescent Mental Health Services (CAMHS), Department of Health, 6009 Perth, Australia; 3 Telethon Kids Institute, 6009 Perth, Australia; 4 Acute Child and Adolescent Mental Health Services (CAMHS), Department of Health, 6009 Perth, Australia; vita.dingerkus@rwth-aachen.de; 5 Clinic for Child and Adolescent Psychiatry, Psychosomatics and Psychotherapy, RWTH Aachen University Hospital, 52074 Aachen, Germany; tilman.gaber@posteo.de (T.J.G); katrin.helmbold@gmail.com (K.H.); sarah-busch@outlook.com (S.B.-B.); becci.koe@gmx.de (R.K.); 6 Department of Ophthalmology, Triemli Hospital Zürich, CH-8063 Zürich, Switzerland; 7 Community Child and Adolescent Mental Health Services (CAMHS), Department of Health, 6009 Perth, Australia; 8 School of Psychological Sciences, Faculty of Science, The University of Western Australia, 6009 Perth, Australia

**Keywords:** neuropeptide Y, acid base status, methionine, tryptophan, serotonin depletion, mental health

## Abstract

Central nervous serotonin (5-HT) can influence behaviour and neuropsychiatric disorders. Evidence from animal models suggest that lowered levels of neuropeptide Y (NPY) may have similar effects, although it is currently unknown whether decreased central nervous 5-HT impact NPY concentrations. Given that the production of NPY is dependent on the essential amino acid methionine (MET), it is imperative to account for the presence of MET in such investigations. Hence, this study sought to examine the effects of acute tryptophan depletion (ATD; a dietary procedure that temporarily lowers central nervous 5-HT synthesis) on serum concentrations of NPY, whilst using the potential renal acid load indicator (PRAL) to control for levels of MET. In a double-blind repeated measures design, 24 adult humans randomly received an AA-load lacking in TRP (ATD) on one occasion, and a balanced control mixture with TRP (BAL) on a second occasion, both with a PRAL of nearly 47.3 mEq of MET. Blood samples were obtained at 90, 180, and 240 min after each of the AA challenges. ATD, and therefore, diminished substrate availability for brain 5-HT synthesis did not lead to significant changes in serum NPY concentrations over time, compared to BAL, under an acute acidotic stimulus.

## 1. Introduction

Central nervous serotonin (5-HT) plays an important role in modulating behaviours. Impairments and changes within this particular neurotransmitter system have been associated with a number of neuropsychiatric disorders, and in particular anxiety and depression [1], and have been documented to impact on behaviours related to hunger and satiety, reproduction and attentional processes [2]. The impact of temporarily lowered central nervous synthesis of 5-HT on human behaviours has been studied in both adults as well as children and adolescents [3] through neurochemical challenge techniques such as acute tryptophan depletion (ATD). ATD is based on the premise that central nervous 5-HT is generated from the amino acid (AA), tryptophan (TRP), which is an essential AA and can only be made available to the body via food intake. Hence, as TRP is only replenished by dietary means, availability of 5-HT in the body (including the central nervous system), diminishes if TRP intake and thus substrate availability for central nervous 5-HT synthesis is reduced. Findings relating to the impact of a diminished central nervous 5-HT synthesis rate on mood and anxiety [1], in particular, have also been in line with the development of selective serotonin reuptake inhibitors (SSRIs) as a pharmacological treatment for patients with mood and anxiety disorders.

Notably, evidence from animal research, pharmacological studies, and trials with humans have suggested that the availability of neuropeptide Y (NPY) may also play an important role in a number of psychiatric disorders, in addition to modulating different aspects of behaviours, such as reproductive behavior and hunger and satiety [4,5]. NPY is a c-terminal amidated peptide and is the most expressed hormone in the arcuate nucleus, but it is also represented in a number of other brain areas, as well as the periphery [6,7]. In humans, NPY contains 36 AAs, including the essential AA methionine (MET) [8]. Importantly, levels of MET in the body are influenced by dietary means. As such, an important implication is that levels of NPY are also reliant on the amount of MET that has been ingested (via protein). There is an abundance of literature suggesting that NPY may be inversely associated with levels of stress, hunger, anxiety, depressed mood [9], alcoholism, and epilepsy [4].

Whilst strong evidence has been described for the relationship between NPY and several neuropsychiatric disorders that are also related to central nervous 5-HT [4], the relationship between NPY and central nervous 5-HT appears to be less clear. Studies relating to eating disorders have shown that 5-HT may regulate appetite and induce satiety in humans [10]. Based on these findings, pharmacological treatments of human obesity have begun targeting the 5-HT system to regulate appetite [11,12]. Other research findings relating to humans have suggested that acute central nervous serotonergic dysfunction (e.g., a short-term decrease in 5-HT synthesis) may impact plasma NPY concentrations, in particular because the release of NPY from sympathetic nerve terminals may be regulated by serotonergic functions [13]. In rodent studies, hypothalamic NPY concentrations were found to increase following an injection of a 5-HT antagonist [14]. In a separate study, two separate groups of mice were administered the SSRI fluoxetine and NPY, and both administrations were associated with decreased immobility times in the forced swim test (FST; often considered as a potential marker of depression-related behaviours in rodent models) compared to a third group that received saline [15]. However, duration of immobility in the FST was not impacted upon in groups of mice that were administered a central nervous 5-HT depletion prior to the administration of either fluoxetine or NPY. With regards to the NPY group, findings suggests that the availability of central nervous 5-HT impacts on the processing of subsequent administration of NPY. Other studies investigating the relationship between central nervous 5-HT and peripheral NPY in humans have examined how the administration of antidepressants may impact on subsequent peripheral NPY concentrations [16]. Another study found a decreased level of plasma NPY concentrations in patients with a major depressive disorder [9]. As the review by Wu et al. suggests, findings have been mixed [4].

Levels of NPY in the human body can be measured whilst controlling for availability of MET, as MET is a core component of the neuropeptide and is also considered to be acidifying. Hence, it is important that MET levels are accounted for when assessing NPY because MET levels are influenced by dietary means. Controlling for levels of MET may ensure a consistent baseline from which NPY may be generated by the body. The potential renal acid load (PRAL) indicator [17] measures the amount of consumed minerals and sulfur-containing proteins [18], and therefore acid–base status can serve as an important parameter to ensure that consistent levels of MET can be determined. Given that MET contains sulfur, it has acidifying properties [19].

To date, investigations into the relationship between central nervous 5-HT and NPY in humans have not accounted for levels of MET. Consequently, this study aimed to investigate whether ATD, a neurochemical challenge procedure that decreases central nervous 5-HT synthesis, was associated with the reduction in peripheral NPY concentrations. To account for levels of MET, an initial body weight adjusted level of MET for all participants was provided as part of the challenge procedure. A reduction in peripheral NPY would be indicated by a smaller release of NPY in the sympathetic nerve terminals. As there is some evidence to suggest that plasma NPY decreases following ATD [13,20], it was hypothesised that ATD would be associated with decreased concentrations of peripheral NPY. In addition, very little is known regarding gender-specific responses to ATD. As such, a secondary aim of the study was to explore the relationship between reduced central nervous 5-HT synthesis (from ATD) and plasma NPY concentrations between genders. As this was an exploratory aim, no explicit hypotheses were formulated. Potential findings in NPY synthesis between genders may be relevant to stress-mediated processes and psychiatric disorders.

## 2. Materials and Methods

### 2.1. Participants

Participants included adults who were in good physical and mental health. The exclusion criteria included: IQ under 85, lacking knowledge of the German language, developmental disorders, schizophrenia, affective disorders, any psycho-organic syndromes, substance abuse, somatic disorders, any regular use of medication (except for hormonal contraceptive intake), and pregnancy. All participants were first screened by an experienced clinician via interview to ensure that participants did not meet the exclusion criteria. The presence of psychiatric disorders were also screened for through the use of the SKIDPIT-light, which is a standardised interview [21]. All the participants provided oral and written informed consent to participate in the study, and were financially compensated after participation in the study [3].

### 2.2. Ethical Approval

The study protocol was approved by the Ethics Committee of the Faculty of Medicine, RWTH Aachen University (Germany), the ethics internal reference number is EK 225/09, and was carried out in accordance with the Helsinki Declaration [3]. 

### 2.3. Study Design

This study utilised a randomized double-blind, within subject repeated measures design. Specifically, the administration of the ATD challenge condition and a control condition served as the within-subject repeated measures factor, and participants were randomized to receive the ATD (Moja-De protocol) [22,23,24,25,26,27,28] and a TRP balanced control mixture (BAL) on two different study days separated by a period of at least seven days. Administration of the AA beverages were also double-blinded, whereby the ATD and BAL AA beverage pairs were indicated by a random number and were provided in brown wide-neck bottles that were non-distinguishable.

### 2.4. Procedures

Participants had an overnight protein fast from 8:00 p.m. the night prior to each of the two study days. Participants were also required to provide confirmation that they had a TRP-free breakfast prior to attending the study day. As mentioned above, the two study days were spaced at least seven days apart. The two study days started between 8 and 9 a.m., where either the ATD or BAL beverages were randomly administered. Total duration of each study day was approximately 4.5 h. 

### 2.5. Biochemical Assessments

On each study day, four blood samples were taken for the assessment of NPY and relevant AA (see [3]). Times points of blood draw were: before the administration of the ATD/BAL beverage at baseline (T0), the second sample was taken after 90 min (T1), the third sample taken after 180 min (T2) and the last sample taken after 270 min (T3). Also at T0, participants undertook drug screening via a urine test. Female participants were additionally required to complete a pregnancy test at this time. T0–T4 also represent the specific time points in which information relating to dependent variables (NPY) were collected. The dependent variables are further described below. 

### 2.6. Depletion Procedure

For this study, ATD was the chosen procedure for the following reasons. Past research has shown that the Moja-De modification of the ATD-test is generally well tolerated, despite a significant reduction in the TRP influx over the blood brain barrier into the central nervous system [22,23,29]. This Moja-De protocol has additional advantages, such as consisting of 7 AA rather than 15, therefore resulting in a reduced AA load, the use of body-weight to determine the dosage of AA, meaning that this protocol can be used in young people and adults, the ability to use this protocol in both human and rodent studies [30], and because this protocol has a validated control AA load (containing TRP), which has not resulted in increased central nervous 5-HT. The overnight protein fast is part of the protocol in order to allow for more standardized conditions for challenge (ATD/BAL) intake. The short-term nature of this protocol is also a benefit, as participants in this study were monitored for the duration in which decreased central nervous 5-HT was most likely to have occurred. The amount of relevant AAs that was contained in the ATD Moja-De beverage was administered in accordance with body weight, in an aqueous suspension. All AAs were provided by the pharmacy of the Faculty of Medicine, RWTH Aachen University. The AA quantities in the ATD Moja-De beverage were as follows (dosage per 10 kg body weight): L-phenylalanine (PHE 1.32 g), L-leucine (LEU 1.32 g), L-isoleucine (ILE 0.84 g), L-methionine (MET 0.5 g), L-valine (VAL 0.96 g), L-threonine (THR 0.6 g), and L-lysine (LYS 0.96 g). The BAL condition/beverage contained the same AA quantities with an additional 0.7 g of TRP per 10 kg of body weight. After each study day, participants were offered a vitamin tablet that is commercially available containing niacin, as niacin is a TRP-derived vitamin.

### 2.7. Laboratory Assessment

Following blood draw, samples were kept at room temperature for 30 minand were then centrifuged at 3500× *g* for 10 min. For this study, serum was collected in a non-heparinised tube for the assessment of the AAs. The plasma was collected from a heparinized tube, and was used to assess all other parameters. Following the preprocessing procedure, the serum and plasma were stored at −80 °C until transportation to the laboratory for analysis.

We used high-pressure liquid chromatography (HPLC) after precolumn derivatization using orthophthaldialdehyde (OPA) to determine the concentrations of the AAs. In order to separate albumin-bound TRP from free TPR, we used an Amicon Ultra-0.5 centrifugal filter set at 14,000× *g* for 30 min (Merck-Millipore, Darmstadt, Germany). This filter retains compounds larger than 10 kDa [3].

The concentration of NPY was assessed by a competitive radio immunoassay (IBL International GmbH, Hamburg, Germany). This process utilized an antiserum with antibodies tagging synthetically to produced NPY which was conjugated to bovine thyroglobulin. The NPY of the test and control samples competed with the 125I-NPY for the binding to these antibodies. NPY concentrations were assessed by the inversely proportional binding of 125I-NPY and the NPY concentration in the test and control samples.

### 2.8. Calculation of TRP Influx

In order to calculate the influx rates for the AA uptake from the plasma into the brain, the Michaelis–Menten equation with a correction for multiple substrate competition was used [31,32]. The rationale behind the use of this equation is evidence-based and builds on the premise that TRP influx across the blood brain barrier is unidirectional in nature, reliant on TRP concentrations and competing LNAAs. Notably, the brain capillary LNAA carrier L-1 is the main transport mechanism for LNAAs, and these cannot be synthesized in the central nervous system (CNS) [31,33,34]. The Michaelis–Menten equation accounts for availability and concentration of a particular substrate, and also provides an approximate reaction rate. As such, the net uptake of TRP across the blood brain barrier is dependent on: first, the passive transport of AAs at L-1 that can be facilitated by integral proteins, and second, a proportion that follows passive diffusion [29]. The Michaelis–Menten formula that was used to calculate the influx-rate of TRP over the blood brain barrier is as follows

$$TRP\;influx=VmaxC/\left(Km\left[1+\sum^{\text{​}}\left(Ci/Ki\;\right)\right]+C\right)+KdC$$

with *C* = plasma concentration of TRP, *Vmax* = maximum rate of conversion, *Km* = affinity constant for *TRP* (Michaelis constant), *Ci* = plasma concentration of CAAs, *Ki* = affinity constants of CAAs, *Kd* = diffusion constant [3,30].

Note that the *Km* (also known as the Michaelis constant) equates to 50% of the *Vmax* for the relevant AA. Additionally, transport constants for different AA from the literature [3,32,34] were used in this mathematical model.

### 2.9. Data Analysis

The data were analysed using SPSS (IBM Software Group, Armonk, NY, USA) and Graph Pad Prism (GraphPad, La Jolla, CA, USA). The influx of TRP was calculated with Excel (Microsoft Corp., Washington, DC, USA).

There were a total of 24 adult participants in this study (aged 21–30 years, mean age 25.3 years, 12 male). Mean weight was 70.54 ± 11.86 kg; the mean BMI was 23.04 ± 1.86 kg/m^2^. The complete characteristics of the study sample relating to the two genders are provided via the supplementary online materials in the paper by Dingerkus et al., (2012) [3]. With regards to the order of AA beverage administration, 9 participants (37.5%, 6 females and 3 males) received the ATD beverage on their first study day, and 15 participants (62.5%, 6 females and 9 males) received ATD on their second day of participation [3]. 

Data were first tested for normality using the Kolmogrov–Smirnov goodness of fit test. This test was applied to the total sample and the male and female groups separately. Separate repeated-measures analyses of variance (RMANOVAs) explored the influence of ATD on NPY concentration, whilst providing an initial body weight adjusted level of MET for all participants. Challenge (ATD vs. BAL) and time after intake (T1–T3) were the within-participant factors, whereas gender was a between-participants factor. The level of statistical significance was set and kept at *p* < 0.05. Because of the exploratory nature of the present investigation, significant *p*-values were not subjected to alpha-adjustment. Tests of normality revealed that all variables were normally distributed except for THR, and VAL concentrations for the female subgroup. Data screening also showed extremely high NPY values for one male participant, leading to exclusion at the data analysis phase. This particular participant was instructed to seek further medical advice because high values of NPY can be associated with phaeochromocytoma, neuroblastoma, or b-cell leukaemia [35,36,37]. As a result of this exclusion, the final analyzed study sample as regards NPY-related data yielded included *N* = 23 healthy adult participants. 

## 3. Results

### 3.1. Potential Renal Acid Load (PRAL)

As specified above, individuals consumed AA beverages that were dosed per 10 kg body weight. Given that the mean body weight was 70.54 ± 11.86 kg, the mean amount of MET consumed was 3.527 ± 0.593 g in both the ATD and the BAL conditions. As the AA beverages that were utilised were not part of a natural diet, the 100% absorption rate was applied in the calculation of PRAL. Consequently, the consumption of this level of MET acutely increased the absorbed acid load by a mean amount of 47.3 ± 7.95 mEq across participants.

### 3.2. NPY Concentrations

Table 1 shows the mean values of the serum NPY concentrations. Overall, on a descriptive level a decreasing trend was observed relating to NPY serum over time, after both ATD and BAL intake and for both male and female participants. Identical results were detected when each gender was considered separately.

#### 3.2.1. Effects of Time and Neurochemical Conditions on NPY Concentrations

For the factor ‘time’ there was a highly significant effect for peripheral NPY concentrations (*F* [2,44] = 11.671; *p* < 0.001), with mean levels of NPY concentration decreasing over time. Concentrations of NPY were not significantly different between neuro-chemical challenge conditions (ATD/BAL). However, significant differences were detected from concentrations of NPY serum concentrations at baseline (T0 = just before ATD/BAL intake) between the two neurochemical challenge conditions (ATD/BAL), with higher mean levels recorded for the day of ATD condition administration. A further RMANOVA was conducted to investigate whether there were any differences between concentrations of NPY after the intake of the respective ATD/BAL AA beverages (T1–T3 time points only). Results of this RMANOVA indicated that there were no significant differences between NPY concentrations following ATD/BAL consumption (*F* [1,88] = 2.423; *p* = 0.135). This suggests that an effect of ATD/BAL on NPY serum concentrations could not be immediately detected, and that the significant differences found in the course of NPY concentrations over time were independent of the ATD/BAL intake.

#### 3.2.2. Interactions

There was no significant interaction between gender and neurochemical condition (ATD/BAL; *F* [1,88] = 0.003; *p* = 0.959). Interactions were also non-significant between gender and time (*F* [2,88] = 0.680; *p* = 0.512), and between gender, time, and condition (*F* [2,88] = 1.169; *p* = 0.321). Combined, these findings suggest that NPY concentrations were not substantially different between neurochemical challenge (ATD/BAL) and genders.

### 3.3. Effects of the Challenge Procedure

The results relating to the effects of the challenge procedure on TRP-influx into the brain have been reported elsewhere [3]. As shown in the paper by Dingerkus et al. (2012), ATD challenge administration led to a significant reduction in TRP-influx over the blood brain barrier into the central nervous system (decreased substrate availability for brain 5-HT synthesis) in a safe and effective manner and was overall well tolerated [3].

## 4. Discussion

The current study aimed to investigate the impact of ATD and subsequently diminished central nervous 5-HT synthesis on peripheral NPY concentrations whilst providing an initial body weight adjusted level of MET for all participants. Having a consistent baseline level of MET was of interest, as generation of NPY is reliant on the essential AA, MET. The rationale for this study was the significant overlap in behaviors that appear to be modulated by both central nervous 5-HT and NPY as demonstrated by evidence from rodent models and human studies. Therefore, it was hypothesized that ATD and the subsequent short-term decrease in central nervous 5-HT synthesis would be associated with decreased peripheral concentrations of NPY in healthy adult humans, compared to the BAL condition. Understanding the relationship between central nervous 5-HT availability and peripheral NPY concentrations is of particular importance with regards to the neurobiology of stress-associated neuropsychiatric disorders, for example anxiety and depressive disorders [38].

Contrary to our expectation, peripheral concentrations of NPY were not significantly different between ATD and BAL, suggesting that levels of NPY were not impacted by a short-term decrease in central nervous 5-HT synthesis (in the ATD condition). However, there may be a number of possible reasons for this finding. For example, it is important to note that a substantial proportion of NPY is produced in the human periphery, and the NPY samples that were used in this study may serve as detection for NPY levels in the periphery, rather than in the central nervous system. Specifically, NPY concentration has been found to be regulated through NPY release from the sympathetic perivascular nerve endings modulated by serotonergic functions [7,20]. Based on the present findings, it could be suggested that an acute central nervous 5-HT depletion may not directly influence the release of NPY from the sympathetic perivascular terminals in healthy participants. 

For this particular study, it may also be possible that the NPY that was analyzed is not entirely referent to NPY that is expressed with neurotransmitters. Specifically, NPY has been shown to have a number of functions in addition to having an impact on mood, stress, and appetite. NPY additionally has anticonvulsant and anti-nociceptive functions [39,40], as well as playing a prominent role in innervating the muscular system and the mucosa [40]. As such, it is co-expressed with other neurotransmitters as well as existing in the mammalian intestines [39,40]. This therefore provides a possible explanation for the lack of differences in NPY levels observed between the ATD and BAL conditions. However, whilst the hypothesized differences were not found, the present study is of value, as it aimed to disentangle the relationship between central nervous 5-HT synthesis and peripheral NPY concentrations in humans, whereas most previous research on this subject was done using animal models [14,15]. In particular, this is the first study that used a body weight adapted ATD protocol to study the impact of decreased central nervous 5-HT synthesis on peripheral NPY concentrations.

Another possible reason for the present findings may be that human NPY serum was assessed in the current study, whilst a large proportion of studies relating to the interactions between 5-HT and NPY have been conducted with animal models. A few human based studies have used cerebrospinal fluid probes to collect NPY data, rather than serum. However, a study by Czermak et al. (2008) [13] utilized plasma NPY concentrations from humans under ATD and BAL conditions. This particular study found no significant differences between healthy control subjects and individuals in remission from depression after ATD/BAL intake. However, this particular study contained a number of limitations, including a significant delay in time between taking NPY samples (i.e., between T0 and 5, 7, and 24 h following ATD/BAL intake). This is notable as the maximum reduction of plasma TRP concentrations is expected to take place between three and five hours after intake [41,42]. As the latter samples of NPY did not correspond with the most significant reduction of plasma TRP concentrations, the relationship between ATD administration and peripheral NPY concentration becomes difficult to evaluate, particularly because longer time periods for obtaining NPY concentrations may allow for repletion and other compensatory effects.

The present study also explored the relationship between an acute decrease of central nervous 5-HT synthesis as impacted by ATD and plasma NPY concentration between genders. The current findings did not show a significant difference in peripheral NPY concentration after ATD or BAL challenge between male and female participants over time. This is of particular importance as literature has suggested that women may be more vulnerable to stress than men [43], and a protective role of NPY in stress-related psychiatric disorders, such as post-traumatic stress disorder (PTSD), has been proposed [38]. Whilst these preliminary findings suggest that there is no difference between genders in peripheral NPY release under acute 5-HT depletion in healthy subjects, further research and replication studies are required.

Several limitations may have impacted on the findings of the study. For example, this study consisted of a relatively small sample that was further reduced following the exclusion of a participant. Future studies with larger samples will be required to further explore the relationship between central nervous 5-HT and NPY in humans. Literature also suggests that the AA Leucine might also be involved in the regulation of appetite [44]; however, its relationship with NPY remains poorly understood. Given that Leucine was a compound of the ATD challenge, further investigation of how Leucine may impact on NPY levels may also benefit the current investigation of how central nervous 5-HT may be related to levels of NPY through the use of the ATD/BAL method. Future studies may also benefit from measuring NPY through the use of cerebrospinal fluid to obtain central nervous NPY, rather than peripheral NPY, although this method is more invasive. Additionally, examining whether ATD impacts NPY under a more alkalized condition may be of interest. Last but not least, there was no investigation of the diet of each participant in the present study (for example a detailed diet history). Given that some diets (e.g., vegan diet) are low in methionine [45], a particular diet may influence the baseline methionine concentration in healthy participants. Therefore, a careful investigation of the nutritional background of each participant seems essential in future studies. 

Notably, the present study only assessed the impact of acute depletion of tryptophan on NPY. It is unclear whether chronic depletion of tryptophan will have a different effect either on central or peripheral NPY. There is evidence to suggest that acute and chronic tryptophan depletion differentially affect central 5-HT_1A_ and 5-HT_2A_ receptor binding in rats [46]. Chronic TRP depletion leading to a dysfunctional serotonergic system has also been shown to affect the pattern of circadian rhythm in rats [47]. Chronic depletion of TRP has also shown to significantly alter serotonin turnover and behaviour in rats [48]. However, to our knowledge, there are no studies that assess the effect of chronic TRP depletion on concentrations of NPY in humans. Further understanding of this relationship may be considered for future research.

## 5. Conclusions

This study investigated how ATD, and hence, lowered synthesis of central nervous 5-HT, may impact on peripheral concentrations of NPY after providing healthy adult participants with body weight adjusted levels of MET. Even after the controlling for the proportion of MET ingested by participants (equating to an average PRAL of 47.3 mEq, and hence, the availability from which NPY could be naturally generated by the body), concentrations of NPY did not differ between the ATD or BAL conditions. Whilst initial interpretation of these findings may suggest that short-term decreases in central-nervous 5-HT are not related to peripheral concentrations of NPY, several notable points must be also considered. This includes the possibility that levels of peripheral NPY may not represent central nervous NPY. A number of limitations may also be noted, such as small sample size, sampling interval, as well as the absence of data obtained under prolonged depletion and which could be the subject of future studies but which also has some methodological limitations. However, accounting for MET availability is important in the consideration of this relationship and provides merit in the neurochemical challenge method that was utilized. 

Although the present study may be considered as an initial investigation into how diminished central nervous 5-HT synthesis may impact on concentrations of peripheral NPY, future studies investigating this interaction in humans is important for several reasons. First, a large proportion of the studies investigating interactions between central nervous 5-HT and peripheral NPY have been conducted with animal models. Second, if the relationship between central nervous 5-HT and peripheral NPY can be established and replicated in humans, novel treatment strategies for neuropsychiatric disorders in which NPY is implicated may be derived. These disorders may include depression, anxiety related disorders, and post-traumatic stress disorder. Essentially, findings showing the contribution of NPY to neuropsychiatric disorders that are also related to disorders modulated by central-nervous 5-HT may give rise to alternate pharmacological treatments. This may be a benefit due to the limitations of SSRIs.

## Figures and Tables

**Table 1:** Mean serum NPY levels over four time points.

Challenge Condition (ATD/BAL)	Gender	Time Point	NPY Concentration (ρg/L)	
		(T)	(M)	(SD)
ATD	Total	0	80.06	20.23
		1	75.51	19.89
		2	70.58	17.4
		3	67.26	16.67
	Men	0	78.89	25.7
		1	74.82	21.8
		2	71.25	20.47
		3	68.58	17.97
	Women	0	81.13	14.68
		1	76.14	18.93
		2	69.98	14.96
		3	66.05	16.08
BAL	Total	0	71.69	14.67
		1	70.86	17.74
		2	65.1	15.26
		3	64.17	15.42
	Men	0	71.41	18.27
		1	71.19	20.53
		2	63.85	18.98
		3	66.67	17.07
	Women	0	71.95	11.26
		1	70.55	15.69
		2	66.24	11.64
		3	61.88	14.08

Means (M) ± standard deviation (SD) for serum NPY concentration (ρg/L) at different time point T0 (Baseline), T1 (90 min), T2 (180 min), and T3 (270 min) after intake of the acute tryptophan depletion (ATD) and the balanced amino acid load (BAL, control condition) challenge.
